# Use of flucinolone acetonide for patients with diabetic macular oedema: patient selection criteria and early outcomes in real world setting

**DOI:** 10.1186/s12886-015-0178-9

**Published:** 2016-01-05

**Authors:** Ibrahim Elaraoud, Walter Andreatta, Andrej Kidess, Ajay Bhatnagar, Marie Tsaloumas, Fahad Quhill, Yit Yang

**Affiliations:** Wolverhampton Eye Infirmary, New Cross Hospital, Wednesfield Road, Wolverhampton, WV10 0QP UK; Queen Elizabeth University Hospital Birmingham, Birmingham, B15 2TH UK; Royal Hallamshire Hospital, Glossop Road, Sheffield, S10 2JF UK; School of Life and Health Sciences, Aston University, Birmingham, B4 7ET UK

**Keywords:** Fluocinolone Acetonide, Iluvien, Diabetic macular oedema, Patient selection, early clinical outcome, visual acuity, central retinal thickness

## Abstract

**Introduction:**

Fluocinolone acetonide slow release implant (Iluvien®) was approved in December 2013 in UK for treatment of eyes which are pseudophakic with DMO that is unresponsive to other available therapies. This approval was based on evidence from FAME trials which were conducted at a time when ranibizumab was not available. There is a paucity of data on implementation of guidance on selecting patients for this treatment modality and also on the real world outcome of fluocinolone therapy especially in those patients that have been unresponsive to ranibizumab therapy.

**Method:**

Retrospective study of consecutive patients treated with fluocinolone between January and August 2014 at three sites were included to evaluate selection criteria used, baseline characteristics and clinical outcomes at 3-month time point.

**Results:**

Twenty two pseudophakic eyes of 22 consecutive patients were included. Majority of patients had prior therapy with multiple intravitreal anti-VEGF injections. Four eyes had controlled glaucoma. At baseline mean VA and CRT were 50.7 letters and 631 μm respectively. After 3 months, 18 patients had improved CRT of which 15 of them also had improved VA. No adverse effects were noted. One additional patient required IOP lowering medication. Despite being unresponsive to multiple prior therapies including laser and anti-VEGF injections, switching to fluocinolone achieved treatment benefit.

**Conclusion:**

The patient level selection criteria proposed by NICE guidance on fluocinolone appeared to be implemented. This data from this study provides new evidence on early outcomes following fluocinolone therapy in eyes with DMO which had not responded to laser and other intravitreal agents.

## Background

Diabetic Macular Oedema (DMO) is one of the leading causes of blindness in the working-age population [1, 2] In the UK, 17.7 % of severe sight impairment certifications in adults aged 16–64 has been attributed to diabetes [3]. Several landmark clinical trials have established the firm position of intravitreal anti-VEGF agents as the first line therapy of choice for DMO [4–6]. Intravitreal corticosteroids have also been shown to be effective in achieving visual gain and reduction of oedema in DMO and with reduced frequency of injections needed [7–9], but with associated risks of causing steroid induced cataract or ocular hypertension and glaucoma. Current guidelines therefore, recommend their use only in those eyes which have either chronic oedema not responsive to laser or those eyes with oedema that have not responded to repeated injections of anti-VEGF agents [10, 11].

There are currently three corticosteroid agents that have been used for DMO; triamcinolone [7], fluocinolone implant releasing 0.2 μg/day of fluocinolone acetonide (ILUVIEN®, Alimera Sciences Limited, Aldershot, UK) [8] and dexamethasone implant containing 700 μg of dexamethasone (Ozurdex®, Allergan, Inc., Irvine, CA, USA) [9]. The United Kingdom was one of the first countries to approve the use fluocinolone implant for treatment of DMO but current National Institute for Care and Health Excellence (NICE) guidance recommends its use only in pseudophakic eyes with DMO that has been unresponsive to other available therapy [10]. Due to this unique selection criteria and early exposure to this technology, the setting in our National Health Service in the United Kingdom is therefore ideal for reporting the implementation of this guidance, in particular, the early experiences in patient selection behaviour by clinicians and also early clinical outcomes in the real world. Such data may be useful for developing future strategies for reducing the frequency of side effects, design of follow-up regimen that can further justify the use of such a unique treatment with its prolonged duration of action.

This retrospective study reports the initial real world experience of implementation of TA301 in the clinical setting, in particular, the patient level criteria used for selection of patients for intravitreal fluocinolone implant, the initial treatment response and the short term safety profile.

## Methods

Consecutive patients treated at each of three hospital departments using fluocinolone implant for DMO according to NICE guidance (TA301) between January 2014 and August 2014 were identified at each site using their respective patient database systems for tracking patients on intravitreal therapies. The study involved collection of data retrospectively from case notes and OCT scans performed as part of patients’ standard care pathways at each institution, approval by Ethics Committee was not required according to the institutional review policies at each site. Case notes review was performed and data were collected at baseline, which was at the time of fluocinolone implant and at the visit nearest to the 3 month time-point of follow-up. At the baseline time-point, data were collected on demographics, visual acuity (treated and fellow eyes), OCT parameters, IOP, IOP lowering medication use, presence of co-existing ocular pathology and prior laser for DMO and DR or intravitreal therapy and duration of DMO. At the 3 month time-point, data were collected on visual acuity (treated eyes), IOP, IOP lowering medication use and OCT parameters. Snellen visual acuity was converted using a standard estimation method so that all acuity data was presented in terms of number of ETDRS letters. The type of conversion method we used would convert 6/60 to 35 letters and 6/6 to 85 letters as obtained when an ETDRS acuity is used at 2 meters for acuity scoring. [12, 13]. Descriptive statistics were used to present the data to demonstrate the spectrum of baseline characteristics with particular emphasis on the types of prior therapies which had been attempted on these eyes prior to using fluocinolone. Short term follow-up data was analysed to show the initial efficacy and occurrences of any serious adverse events.

## Results

A total of 22 patients (14 M:8 F) aged between 42 and 85 years (mean 67.2 years) were included. Nineteen patients had Type-2 and 3 patients had Type-1 diabetes mellitus; mean duration of diabetes was 17.9 years (range: 3–60). All patients had unilateral fluocinolone implants ie; 11 right and 11 left eyes treated.

Visual acuity conversion for Snellen fraction to ETDRS was required for five patients. In all other patients, acuity scores were captured directly using ETDRS chart. Treated eyes had mean baseline visual acuity of 50.7 letters and mean CRT of 631 μm. Twenty two eyes had foveal eversion and 13 eyes had pre-existing vitreo-retinal interface abnormalities. Majority of eyes had prior laser of either macular or pan retinal photocoagulation. All 22 eyes had prior intravitreal therapy. Thirteen eyes had prior ranibizumab or bevacizumab, 3 eyes had both ranibizumab and bevacizumab and 6 eyes had prior intravitreal triamcinolone for DMO. Mean duration of DMO was 26.2 months (range: 9-47). All patients had a minimum of 8 weeks wash out period after using Anti-VEGF before having the fluocinolone implant.

Fellow eyes had visual acuities ranging from NPL to 85 letters. In 11 patients, the fellow eye had visual acuities equal to or worse than treated eyes. Baseline characteristics of 22 treated eyes are shown in Table 1. From these characteristics, it is evident that the eyes that were selected had moderately low visual acuities, severe macular thickening, presence of other co-existing pathologies such as epiretinal membranes and controlled glaucoma.

**Table 1:** Baseline parameters of Fluocinolone Acetonide implant treated eyes

Parameter	Value
Visual Acuity (ETDRS letters)	Range (2–85), Mean (50.7)
Central Retinal Thickness (Micrometers)	Range (373–1052) .Mean (631)
Duration of DMO(Months)	Range (9-47). Mean (26.2)
Foveal eversion	21
Pseudophakia	22
Eyes with one co-existing pathology	ERM(n = 11)
	VMT(n = 2)
	Glaucoma/Ocular hypertension (n = 4)
	NVD n = 1
	NVE n = 1
Eyes with >1 co-existing pathologies	3
Eyes on IOP lowering drugs	4
Eyes with prior macular laser only	15
Eyes with prior vitrectomy for DMO/ERM	1
Eyes with prior intravitreal therapy	Ranibizumab only– n = 11
	Bevacizumab only n = 2
	Rani and Beva n = 3
	Triamcinolone n = 6
Eyes with prior PRP	9

At the 3 month time-point, mean change in BCVA for all patients was +6.4 ETDRS letters (SD: ±7.2; range: -11 to +25). A gain of >0 letters was seen in 21 eyes (95.4 %); 0-4 letters in 4 eyes (18.1 %), 5-9 letters in 7 eyes (31.8 %) and ≥10 letters in 6 eyes (27.2 %), CRT after three months reduced by 148.9 μm (SD: ±240.6; range: -714 to +385 μm). A total of 18 out of 22 eyes (81.8 %) showed some reduction in CRT at 3-months. In the 4 eyes without CRT improvement, the mean increase in CRT was 134.75 μm (SD: ±169.8; range: +26 to +385 μm).

Overall, 15 patients (68.2 %) showed some improvement in both visual acuity and CRT, 3 patients (13.6 %) showed some visual gain without any reduction in CRT, another 3 patients (13.6 %) achieved a reduction in CRT without any corresponding visual gain and 1 patient (4.5 %) did not show any improvement in visual acuity or CRT.

Of the 5 eyes which had undergone prior vitrectomy, visual acuity was improved by +7.2 letters (range: 0 to +14) and CRT reduced by 176.8 (range:-714 to +385). Four of 5 eyes showed both a reduction in CRT with improved visual acuity but one patient had reduced CRT (-345) with no gain in visual acuity.

One patient worsened by 11 letters and had increase in CRT of 385 μm; this particular patient had received prior treatment with macular laser and four ranibizumab and one triamcinolone intravitreal injections.

Mean baseline IOP was 16.9 mmHg (SD: ±3.1; range 10–22 mmHg), with the mean change of 0.3 mmHg (SD: ±3.1; range: -7 to +5 mmHg) at month 3. No cases with substantial elevation in IOP were documented in the short follow up period. Of 22 eyes, four eyes, were receiving IOP-lowering drops (timolol and/or latanoprost) prior to fluocinolone implant. Following implant an additional one eye needed IOP lowering medication.

## Discussion

Our study set out to evaluate two important aspects in the use of fluocinolone acetonide in the real world clinical setting for treatment of DMO; firstly, the characteristics of patients selected for treatment by clinicians and secondly, the early treatment response seen in their patients.

In terms of selection, we found that fluocinolone implant was used infrequently in patients with DMO. In all three sites, there were only 22 procedures performed in three sites in the 8-month period following approval of fluocinolone by NICE. Given the large number of patients needing repeated injections of ranibizumab for DMO at these sites [14], this infrequent use of fluocinolone suggests that clinicians had a high threshold for using fluocinolone for DMO. The majority of patients did have severe oedema with mean CRT of over 600 μm. The majority of eyes had prior macular laser and all eyes had prior intravitreal therapy. This finding supports the fact that clinicians are following the NICE guidance criterion of unresponsiveness to other available therapy [10]. Six eyes had prior intravitreal triamcinolone but no anti-VEGF. This reflects the general acceptability of switching between corticosteroid agents especially if prior triamcinolone did not cause uncontrolled glaucoma [15]. This may be an observation of a growing trend to use a shorter acting intravitreal corticosteroid such as triamcinolone as a steroid challenge in these patients, a trend which has been adopted by the American Food and Drug Administration (FDA) when fluocinolone was approved to be used in DMO [11]. Another criterion stipulated by NICE guidance is that the treated eyes had to be pseudophakic. In this survey, all treated eyes were already pseudophakic indicating that clinicians are following NICE guidance recommendation carefully. We were also interested to evaluate whether clinicians were reluctant to use fluocinolone in better seeing eyes. With 11 out of 22 eyes being the better seeing eye, it does not appear to be the case that there was any reluctance in the selection behaviour of clinicians to avoid fluocinolone in better seeing eyes. However the majority of eyes had foveal eversion on OCT. This may be a future additional criterion of severity of oedema that could be used to select the most justifiable cases for fluocinolone therapy. All patients in this study had chronic DMO of more than 2 years, Authors could not identify any particular pattern between chronicity of DMO and early response/failure of this cohort of patients although admittedly is a short follow up period.

**Fig. 1:**
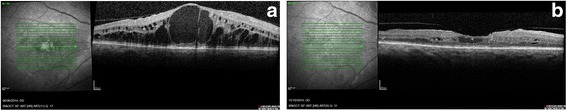
**a** Spectral Domain OCT at baseline showing extensive intraretinal fluid with foveal eversion. **b** Spectral Domain OCT at three months showing significant resolution of intraretinal fluid and the re-formation of the foveal dip

In terms of early outcome, we found promising results with 18 out of 22 eyes reported to have visual gain and 18 out of 22 eyes with at least a reduction in CRT at month 3 time-point. Figure 1 shows a dramatic reduction of CRT from 960 μm to 246 μm 3 months post fluocinolone. Given the majority of patients had prior injections of ranibizumab or bevacizumab in this study, this highlights the additional value that can be gained by switching from anti-VEGF to intravitreal fluocinolone in unresponsive cases. This also serves as new evidence for switching from ranibizumab to fluocinolone as some patients in the FAME trial had rescue bevacizumab but not ranibizumab and there have been no published series to our knowledge of visual outcome when switching from ranibizumab to fluocinolone. In one case report of a single patient, with longstanding DMO which was unresponsive to ranibizumab, Bertelmann and Shulze reported reduction in oedema and gain in visual acuity following a switch from ranibizumab to fluocinolone implant [16]. The three month follow-up period is admittedly too short for evaluating the risk of IOP rise. In this study, only one patient required commencement of IOP lowering medication at month 3 time-point. Based on available FAME data on IOP response, it is likely that more patients will subsequently develop elevated IOP.

There were limitations in the design of this real world study. The selection of patients for therapy is a complex process involving careful explanation and the confidence expressed by the treating clinician at counselling and a final decision made with the patient preference. It was possible that many other patients who were eligible for therapy but declined due to the risk factors involved. We could not capture those patients who declined treatment with our retrospective design and this study may represent the highest threshold for using fluocinolone at this early stage of implementing NICE guidance. We also captured basic baseline data on patient selection. We were unable in the scope of this study to capture the timescale and progression of DMO in these patients. We recognise that other factors, including the recent worsening of oedema or visual acuity and the frequency of previous visits do have an impact on tendency to use fluocinolone and these factors were not be captured uniformly in this retrospective design.

The strength of this study was the accuracy of the representation of those patients who were treated with fluocinolone. The sites selected were medium sized district general hospital settings as well as large teaching hospital centres which enables translation of our results to the majority of hospital eye departments. The inclusion of all consecutively treated patients in an 8-month period ensured a representative cohort of patients for reporting of a potentially wide spectrum of selection criteria used or variable response to treatment.

## Conclusion

Numerous review articles have been published mentioning fluocinolone therapy for DMO but there is a paucity of published data on use of fluocinolone 0.2 μg/day or iluvien® outside the experience of the FAME trial [8, 17]. We are also unaware of any previously published case series describing the experience of switching from ranibizumab to fluocinolone. This study can hope to serve this small gap in our evidence base and also be of value for designing selection and follow-up protocols for treatment of patients with the fluocinolone implant.

## Abbreviations

DMO: Diabetic macular oedema
VA: Visual acuity
CRT: Central retinal thinkness
Anti-VEGF: Anti-(vascular endothelial growth factor)
NICE: National Institute for Health and Care Excellence
IOP: Intra ocular pressure
OCT: Ocular coherence tomography
EDTRS: Early treatment diabetic retinopathy study
